# JAK2 Inhibitor, Fedratinib, Inhibits P-gp Activity and Co-Treatment Induces Cytotoxicity in Antimitotic Drug-Treated P-gp Overexpressing Resistant KBV20C Cancer Cells

**DOI:** 10.3390/ijms23094597

**Published:** 2022-04-21

**Authors:** Yunmoon Oh, Jin-Sol Lee, Ji Sun Lee, Jae Hyeon Park, Hyung Sik Kim, Sungpil Yoon

**Affiliations:** School of Pharmacy, Sungkyunkwan University, 2066 Seobu-ro, Jangan-gu, Suwon 16419, Korea; oym9083@g.skku.edu (Y.O.); jinsol3361@skku.edu (J.-S.L.); leejs7186@naver.com (J.S.L.); sky3640@naver.com (J.H.P.); hkims@skku.edu (H.S.K.)

**Keywords:** JAK2, fedratinib, co-treatment, cancer, P-gp, drug-resistance

## Abstract

P-glycoprotein (P-gp) overexpression is one of the major mechanisms of multidrug resistance (MDR). Previously, co-treatment with Janus kinase 2 (JAK2) inhibitors sensitized P-gp-overexpressing drug-resistant cancer cells. In this study, we assessed the cytotoxic effects of JAK2 inhibitor, fedratinib, on drug-resistant KBV20C cancer cells. We found that co-treatment with fedratinib at low doses induced cytotoxicity in KBV20C cells treated with vincristine (VIC). However, fedratinib-induced cytotoxicity was little effect on VIC-treated sensitive KB parent cells, suggesting that these effects are specific to resistant cancer cells. Fluorescence-activated cell sorting (FACS), Western blotting, and annexin V analyses were used to further investigate fedratinib’s mechanism of action in VIC-treated KBV20C cells. We found that fedratinib reduced cell viability, increased G2 arrest, and upregulated apoptosis when used as a co-treatment with VIC. G2 phase arrest and apoptosis in VIC–fedratinib-co-treated cells resulted from the upregulation of p21 and the DNA damaging marker pH2AX. Compared with dimethyl sulfoxide (DMSO)-treated cells, fedratinib-treated KBV20C cells showed two-fold higher P-gp-inhibitory activity, indicating that VIC–fedratinib sensitization is dependent on the activity of fedratinib. Similar to VIC, fedratinib co-treatment with other antimitotic drugs (i.e., eribulin, vinorelbine, and vinblastine) showed increased cytotoxicity in KBV20C cells. Furthermore, VIC–fedratinib had similar cytotoxic effects to co-treatment with other JAK2 inhibitors (i.e., VIC–CEP-33779 or VIC–NVP-BSK805) at the same dose; similar cytotoxic mechanisms (i.e., early apoptosis) were observed between treatments, suggesting that co-treatment with JAK2 inhibitors is generally cytotoxic to P-gp-overexpressing resistant cancer cells. Given that fedratinib is FDA-approved, our findings support its application in the co-treatment of P-gp-overexpressing cancer patients showing MDR.

## 1. Introduction

Antimitotic drugs, e.g., paclitaxel, docetaxel, vincristine (VIC), vinorelbine, vinblastine, and eribulin, inhibit mitosis by preventing the polymerization or depolymerization of cell microtubules [1,2,3]. However, their efficacy is limited by the occurrence of multidrug resistance (MDR). It is therefore essential, in the development of novel treatments, that the mechanism(s) underlying cellular cytotoxicity in antimitotic drugs be determined.

P-glycoprotein (P-gp) is a component of the cellular membrane that expels antimitotic drugs and its overexpression is one of the major mechanisms of MDR [4,5,6]. Although P-gp inhibitors have been developed, their toxicity to normal cells has limited their success in clinical tests [4,5,7]. In addition, P-gp inhibitors can alter the pharmacokinetic profile of co-treated drugs and lead to severe side effects [8]. Several attempts have been made to improve their safety for further clinical trials [5,9]. In continuation of these efforts, the present study assessed the efficacy of novel repositioned P-gp inhibiting drugs and their cytotoxic mechanisms in P-gp overexpressing drug-resistant cancer cells. The efficacy of pharmacological treatments for P-gp-overexpressing resistant cancers can be ascertained by identifying novel mechanisms of repositioned drugs, which promotes the approval of these treatments. Repositioned drugs that can increase cytotoxicity to P-gp-overexpressing resistant cancer cells have been reported [10,11,12].

Janus kinase 2 (JAK2) is a protein that contributes to drug-resistant cancer and its inhibition can increase cytotoxicity of chemotherapy in MDR cells [13,14,15,16] and increase the efficacy of anticancer drugs [17,18]. JAK2 inhibitors have been shown to increase drug-cytotoxicity in P-gp-overexpressing drug-resistant cancer cells through P-gp inhibitory activity [19,20,21]. Identifying the mechanisms underlying the effects of various JAK2 inhibitors will promote their clinical application.

This study evaluated the cytotoxic and P-gp inhibitory activity of fedratinib, a recently FDA-approved JAK2 inhibitor [22,23], in P-gp-overexpressing MDR cancer cells. Fedratinib showed P-gp inhibitory activity, which increased early apoptosis in antimitotic drug-treated P-gp-overexpressing resistant KBV20C cancer cells. Our study provides a basis for the development of JAK2 inhibitor-based therapies for drug-resistant cancers.

## 2. Results

### 2.1. Single Treatment of Fedratinib Displays Similar Cytotoxic Effects in Drug-Sensitive Parent KB and Drug-Resistant KBV20C Cells

**Figure 1:**
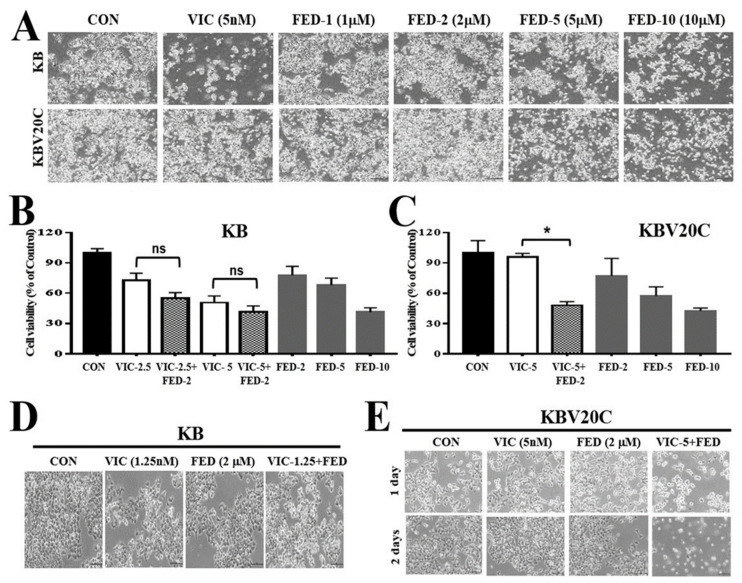
Co-treatment with fedratinib specifically increases the sensitization of drug-resistant KBV20C cancer cells to VIC treatment. (**A**) Parent sensitive KB cells and drug-resistant KBV20C were grown on 60 mm diameter dishes and treated with 5 nM vincristine (VIC), 1 µM fedratinib (FED-1), 2 µM fedratinib (FED-2), 5 µM fedratinib (FED-5), 10 µM fedratinib (FED-10), or 0.1% DMSO (CON). After 1 day, all cells were observed using an inverted microscope at ×40 magnification. (**B**,**C**) Parent sensitive KB cells and drug-resistant KBV20C were plated on 96-well plates and grown to 30–40% confluence. The cells were then treated for 48 h with 2.5 nM vincristine (VIC-2.5), 5 nM vincristine (VIC-5), 2 µM fedratinib (FED-2), 5 µM fedratinib (FED-5), 10 µM fedratinib (FED-10), 10 µM fedratinib (FED-10), 2.5 nM VIC with 2 µM fedratinib (VIC-2.5 + FED-2), 5 nM VIC with 2 µM fedratinib (VIC-5 + FED-2), or 0.1% DMSO (CON). Cell viability assay was performed as described in the Materials and Methods. The data are presented as the mean ±SD from two independent experiments repeated in triplicate. For co-treatments, significantly different at * *p* < 0.05 compared with the corresponding control. The “ns” is an abbreviation of “not significant”. (**D**,**E**) Parent sensitive KB cells and drug-resistant KBV20C were grown on 60 mm-diameter dishes and treated with 1.25 nM vincristine (VIC-1.25), 5 nM vincristine (VIC-5), 2 µM fedratinib (FED), 1.25 nM VIC with 2 µM fedratinib (VIC-1.25 + FED), 5 nM VIC with 2 µM fedratinib (VIC-5 + FED), or 0.1% DMSO (CON). After 1 day, all cells were observed using an inverted microscope at ×100 magnification.

First, we compared the cytotoxic effect of fedratinib on drug-resistant KBV20C cells and drug-sensitive parent KB cells. In a cell viability assay, IC50 values of 6.9 µM for KBV20C and 8.6 µM for KB cells were observed after 48 h of treatment, which indicates similar toxic effects of fedratinib in resistant KBV20C and sensitive KB cells (Figure 1A). From our microscopic observations (Figure 1A), single treatment with fedratinib had cytotoxic effects in both resistant KBV20C and sensitive KB cells, suggesting that fedratinib is not a substrate for P-gp overexpressed on the cell membranes of resistant KBV20C cells. Therefore, it can be used to increase cytotoxicity in P-gp-overexpressing resistant cancer cells. VIC is an antimitotic drug that is routinely used as a chemotherapeutic agent for leukemia, neuroblastoma, and small cell lung cancer [1,2,3,24]. Previously, we showed that KBV20C-resistant cancer cells present a VIC-resistant phenotype through P-gp overexpression [25,26]. VIC only induced cytotoxic effects in KB cells, whereas KBV20C cells showed a resistant phenotype at the same dose (Figure 1A).

### 2.2. VIC–Fedratinib Co-Treatment Increases Cytotoxicity in Drug-Resistant KBV20C Cancer Cells

We tested whether fedratinib at low doses could increase cytotoxicity in VIC-treated KBV20C cancer cells. Co-treatment with fedratinib reduced the proliferation of VIC-treated KBV20C cells when compared with a single treatment with either VIC or fedratinib (Figure 1C).

In comparing the cytotoxic effect of fedratinib co-treatment in drug-resistant KBV20C cells and drug-sensitive parent KB cells, KB cells displayed a smaller degree of sensitization to VIC (Figure 1B,C). These results indicate that low-dose fedratinib is able to specifically sensitize P-gp-overexpressing VIC-resistant KBV20C cells, while having little effect on VIC-sensitive parent KB cells. We confirmed similar sensitization effects between resistant KBV20C and sensitive KB cells through microscopic observations (Figure 1D,E). Neither KB nor KBV20C cells displayed a cytotoxic response to individual treatment with 2 µM fedratinib, and only the parent KB cells had a cytotoxic response to 5 nM VIC (Figure 1B–E). These findings indicate that a low dose of fedratinib can increase cytotoxicity in VIC-treated resistant KBV20C cells.

### 2.3. VIC–Fedratinib Co-Treatment Increases Apoptosis in KBV20C Cells in a Dose-Dependent Manner

**Figure 2:**
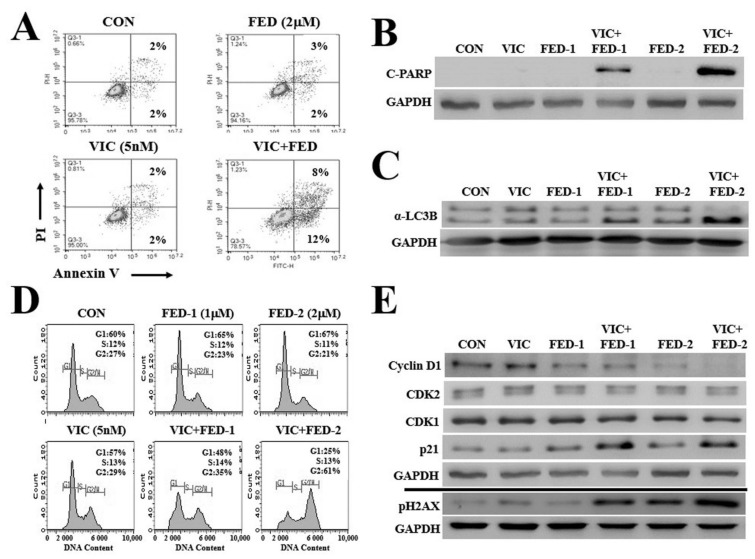
DNA damage signal induces apoptosis and autophagy via G2 arrest in VIC–fedratinib co-treated KBV20C cells. (**A**) KBV20C cells were grown on 60 mm-diameter dishes and treated with 5 nM vincristine (VIC), 2 µM fedratinib (FED), 5 nM VIC with 2 µM fedratinib (VIC + FED), or 0.1% DMSO (CON). After 24 h, annexin V analyses were performed as described in Materials and Methods. (**B**,**C**) KBV20C cells were plated on 60 mm-diameter dishes and treated with 5 nM vincristine (VIC), 1 µM fedratinib (FED-1), 2 µM fedratinib (FED-2), 5 nM VIC with 1 µM fedratinib (VIC + FED-1), 5 nM VIC with 2 µM fedratinib (VIC + FED-2), or 0.1% DMSO (CON). After 24 h, western blot analysis was performed using antibodies against C-PARP, α-LC3B, and GAPDH. (**D**) KBV20C cells were plated on 60 mm-diameter dishes and treated with 5 nM vincristine (VIC), 1 µM fedratinib (FED-1), 2 µM fedratinib (FED-2), 5 nM VIC with 1 µM fedratinib (VIC + FED-1), 5 nM VIC with 2 µM fedratinib (VIC + FED-2), or 0.1% DMSO (CON). After 24 h, FACS analyses were performed as described in Materials and Methods. (**E**) KBV20C cells were plated on 60 mm-diameter dishes and treated with 5 nM vincristine (VIC), 1 µM fedratinib (FED-1), 2 µM fedratinib (FED-2), 5 nM VIC with 1 µM fedratinib (VIC + FED-1), 5 nM VIC with 2 µM fedratinib (VIC + FED-2), or 0.1% DMSO (CON). After 24 h, western blot analysis was performed using antibodies against Cyclin D1, CDK2, CDK1, p21, pH2AX, and GAPDH.

**Figure 3:**
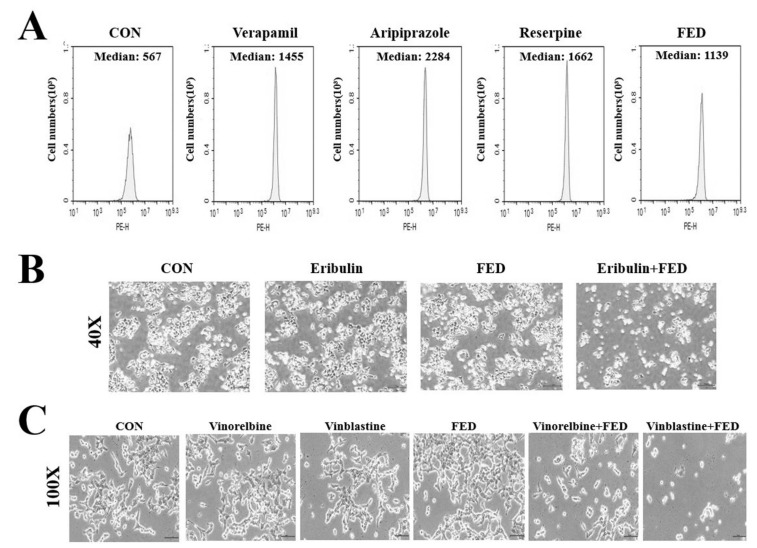
Fedratinib with P-gp inhibitory activity increases the sensitization of KBV20C cells treated to other anti-mitotic drugs. (**A**) KBV20C cells were grown on 60 mm-diameter dishes and treated with 10 µM verapamil, 2.5 µM aripiprazole, 5 µM reserpine, 2 µM fedratinib (FED), or 0.1% DMSO (CON). After 1 h, all cells were stained with rhodamine for 3 h and examined by using FACS analysis, as described in Section 4, Materials and Methods. (**B**,**C**) KBV20C cells were grown on 60 mm diameter dishes and treated with 30 nM eribulin, 0.1 µg/mL vinorelbine, 5 nM vinblastine, 2 µM fedratinib (FED), 30 nM eribulin with 2 µM fedratinib (Eribulin + FED), 0.1 µg/mL vinorelbine with 2 µM fedratinib (Vinorelbine + FED), 5 nM vinblastine with 1 µM fedratinib (Vinblastine + FED), or 0.1% DMSO (CON). After 1 day, all cells were observed using an inverted microscope at x40 or x100 magnification.

We clarified the mechanism of action of VIC–fedratinib co-treatment by apoptotic analysis using annexin V staining. We found that the proportion of early-phase apoptotic cells was approximately 1.5-fold greater than that of late-phase apoptotic cells (Figure 2A), suggesting that the induction of early apoptosis results in the cytotoxic effects of VIC–fedratinib co-treatment. To confirm increased apoptosis in co-treated cells, we measured the levels of a relevant molecular marker, c-PARP. c-PARP expression increased in VIC–fedratinib co-treated cells (Figure 2B). According to a densitometric analysis, c-PARP levels were 25-fold higher in co-treatments than in single treatments. When we compared the c-PARP expression in cells treated with different concentrations of fedratinib (between 1 µM and 2 µM), we found that c-PARP expression increased in a dose-dependent manner in VIC-treated resistant KBV20C cells, while its expression did not change by single treatment with either fedratinib or VIC (Figure 2B). This indicates that fedratinib with low doses is highly effective when combined with VIC treatment in P-gp overexpressing resistant KBV20C cancer cells and ineffective as a monotherapy. Collectively, we demonstrated that fedratinib can specifically increase cytotoxicity in P-gp overexpressing resistant KBV20C cells through the early apoptotic pathway when treated in combination with VIC. We also evaluated autophagic induction in VIC–fedratinib co-treatment. Resistant cancer cells inhibit autophagy, which results in increased cancer cell survival and proliferation [27]. To identify autophagy induced by VIC–fedratinib, we analyzed the degradation of α-LC3B, a protein marker of autophagosome formation [27], by western blot analysis. VIC–fedratinib increased α-LC3B degradation compared with single treatments of VIC or fedratinib (Figure 3C). By densitometric analysis, we determined that α-LC3B levels were two-fold higher in co-treatments than in single treatments. This suggests that VIC–fedratinib co-treatment induced autophagy in drug-resistant KBV20C cells.

### 2.4. VIC–Fedratinib Co-Treatment Induces G2-Arrest and Increases DNA Damage in KBV20C Cells

According to a fluorescence-activated cell sorting (FACS) analysis, VIC–fedratinib co-treatment increased the number of cells in G2 arrest compared with single treatments of VIC or fedratinib (Figure 2D); the proportion of VIC-treated cells in G2 arrest increased in a fedratinib-dose-dependent manner.

We assessed the expression of proteins involved in G2 arrest [28,29] by Western blot analysis. There were no qualitative differences in cyclin protein expression levels between VIC–fedratinib co-treatment and single treatments with either agent (Figure 2E). A slight increase in p21 expression level was detected in cells co-treated with 1 µM or 2 µM fedratinib (Figure 2E). The expression of pH2AX, a DNA damage marker, was upregulated in a dose-dependent manner following co-treatment (Figure 2E), suggesting that DNA damage may increase G2 arrest in VIC–fedratinib co-treated KBV20C cells. We determined by densitometric analysis that pH2AX levels were six-fold higher in co-treatments than in single treatments. We propose that the DNA-damage signal increased early apoptosis via G2 arrest in VIC–fedratinib co-treated KBV20C cells.

### 2.5. Fedratinib Demonstrates P-gp Inhibiting Activity

Thereafter, we assessed whether P-gp inhibition by fedratinib is responsible for its observed cytotoxic effects in VIC-treated KBV20C cells. Known P-gp inhibitors that inhibit P-gp substrate efflux (i.e., verapamil, aripiprazole, and reserpine) were used as positive controls [30,31], and rhodamine 123, a P-gp substrate, was used to measure P-gp inhibition [30,32,33]. In this experiment, yellow cellular fluorescence was indicative of intracellular accumulation of rhodamine 123. Fedratinib increased P-gp inhibitory activity in KBV20C cells by two-fold compared with DMSO-treated controls (Figure 3A), suggesting that P-gp inhibition by fedratinib plays a key role in the cytotoxic effect of VIC–fedratinib co-treatment. KBV20C cells treated with verapamil, aripiprazole, or reserpine (positive controls) showed three- to four-fold higher P-gp inhibitory activity than DMSO-treated cells (Figure 3A). Considering that, even with comparatively lower P-gp-inhibitory activity, fedratinib still sensitized KBV20C cells to VIC, we suggest that fedratinib induces cytotoxicity in VIC-treated KBV20C cells via both P-gp inhibition and other stimulating mechanisms.

### 2.6. Fedratinib Treatment Complements the Cytotoxic Effects of Other Antimitotic Drugs in KBV20C Cells

We also investigated whether fedratinib was effective in combination with other antimitotic drugs. The KBV20C cell line is a useful model of highly-resistant cancer cells [33,34,35]. We previously showed that the concentration of eribulin required for the induction of a similar rate of apoptosis in resistant KBV20C cells was approximately 500-fold greater than that in the parental drug-sensitive KB cells [33,34]. Treatment with 2 µM fedratinib produced greater cytotoxic effects on cells co-treated with eribulin (Figure 3B). The results confirmed that eribulin–fedratinib was as effective as VIC–fedratinib in inducing cytotoxicity in drug-resistant KBV20C cancer cells. This also suggests that fedratinib can be used at a low dose to increase cytotoxicity of eribulin-resistant cancer cells.

**Figure 4:**
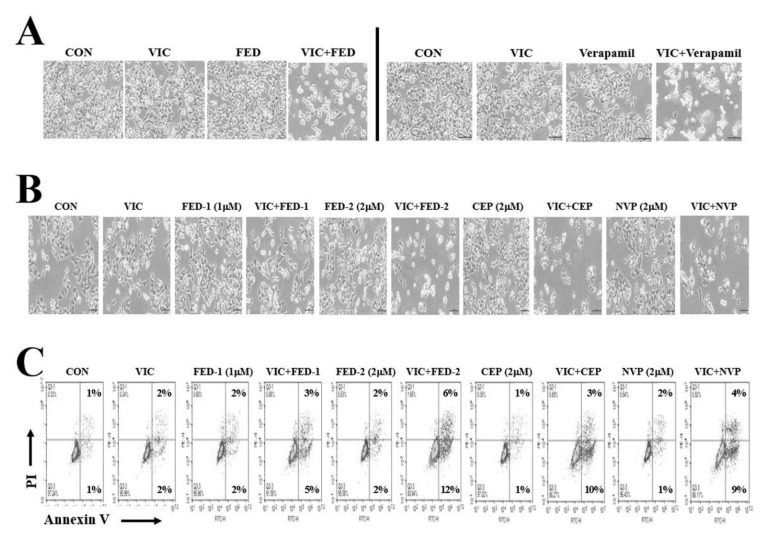
JAK2 inhibitors (fedratinib, CEP-33779, and NVP-BSK805) sensitize VIC-treated KBV20C cells via similar mechanisms of action. (**A**) KBV20C cells were plated on 60 mm diameter dishes and treated with 5 nM vincristine (VIC), 2 µM fedratinib (FED), 10 µM verapamil, 5 nM VIC with 2 µM fedratinib (VIC + FED), 5 nM VIC with 10 µM verapamil (VIC+VER), or 0.1% DMSO (CON). After 1 day, all cells were observed using an inverted microscope at ×100 magnification. (**B**,**C**) KBV20C cells were plated on 60 mm diameter dishes and treated with 5 nM vincristine (VIC), 1 µM fedratinib (FED-1), 2 µM fedratinib (FED-2), 2 µM CEP-33779 (CEP), 2 µM NVP-BSK805 (NVP), 5 nM VIC with 1 µM fedratinib (VIC + FED-1), 5 nM VIC with 2 µM fedratinib (VIC + FED-2), 5 nM VIC with 2 µM CEP-33779 (VIC + CEP), 5 nM VIC with 2 µM NVP-BSK805 (VIC + NVP), or 0.1% DMSO (CON). After 24 h, microscopic observation at x100 magnification (**B**) or annexin V analyses (**C**) were performed as described in Section 4, Materials and Methods.

We tested the cytotoxic effect of fedratinib in combination with vinorelbine and vinblastine, antimitotic drugs that are routinely used as chemotherapeutic cancer agents [2,24]. Vinorelbine–fedratinib and vinblastine–fedratinib co-treatments showed greater cytotoxic effects in P-gp-overexpressing resistant KBV20C cells compared with single treatments with either vinorelbine or vinblastine (Figure 3C). This result suggests that fedratinib could be combined with other antimitotic drugs to increase cytotoxicity in P-gp overexpressing resistant cancer cells. Thereafter, we compared the cytotoxic effects of verapamil with that of fedratinib on VIC-treated KBV20C cells. As shown in Figure 4A, 2 µM of fedratinib and 10 µM verapamil produced similar cytotoxic effects in cells co-treated with VIC, suggesting that lower concentrations of fedratinib induce similar cytotoxic effects to verapamil in VIC-treated KBV20C cells.

### 2.7. JAK2 Inhibitors (Fedratinib, CEP-33779, and NVP-BSK805) Increase Cytotoxicity in VIC-Treated KBV20C Cells through Similar Mechanisms of Action

Fedratinib, CEP-33779, and NVP-BSK805 demonstrated P-gp inhibitory activity and cytotoxic effects in previous studies of VIC co-treated P-gp-overexpressing resistant cancer cells (Table 1) [16,19,20,21,36,37,38]. We compared the cytotoxic effect of these JAK2 inhibitors at the same dose according to IC50 estimates. As seen in Figure 4B, 2 µM of fedratinib, CEP-33779, or NVP-BSK805 showed similar cytotoxic effects in VIC-treated KBV20C cells.

To determine their mechanisms of action, we compared the degree of apoptosis induced by fedratinib, CEP-33779, and NVP-BSK805 on VIC-treated KBV20C cells. As seen in Figure 2A and Figure 4C, co-treatment with VIC–fedratinib, VIC–CEP-33779, and VIC–NVP-BSK805 showed similar increases in rates of early apoptosis. Furthermore, 2 µM of fedratinib was more effective than 1 µM (Figure 2A,B and Figure 4C), suggesting a dose-dependent relationship between JAK2 inhibitors and rates of early apoptosis in VIC co-treated KBV20C resistant cells. These findings indicate that co-treatment with any of these JAK2 inhibitors can increase cytotoxicity in P-gp-overexpressing resistant cancer cells by inducing early apoptosis.

**Figure 5:**
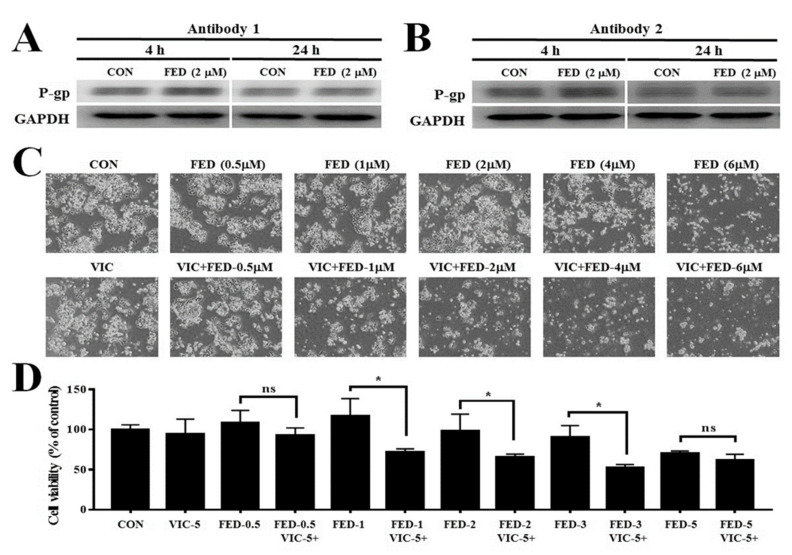
Fedratinib did not change P-gp protein levels. (**A**,**B**) KBV20C cells were plated on 60 mm diameter dishes and treated with 2 µM fedratinib (FED-2) or 0.1% DMSO (CON). After 4 h or 24 h, Western blot analysis was performed using antibodies against (**A**) P-gp (Antibody 1 from Cell signaling company), (**B**) P-gp (Antibody 2 from Santia Cruz company), and GAPDH. (**C**) Drug-resistant KBV20C were grown on 60 mm diameter dishes and treated with 5 nM vincristine (VIC), 0.5 µM fedratinib (FED-0.5), 1 µM fedratinib (FED-1), 2 µM fedratinib (FED-2), 4 µM fedratinib (FED-4), 6 µM fedratinib (FED-6), 5 nM VIC with 0.5 µM fedratinib (VIC-5 + FED-0.5), 5 nM VIC with 1 µM fedratinib (VIC-5 + FED-1), 5 nM VIC with 2 µM fedratinib (VIC-5 + FED-2), 5 nM VIC with 4 µM fedratinib (VIC-5 + FED-4), 5 nM VIC with 6 µM fedratinib (VIC-5 + FED-6), or 0.1% DMSO (CON). After 2 days, all cells were observed using an inverted microscope at x40 magnification. (**D**) Drug-resistant KBV20C were plated on 96-well plates and grown to 30–40% confluence. The cells were then treated with 5 nM vincristine (VIC-5), 0.5 µM fedratinib (FED-0.5), 1 µM fedratinib (FED-1), 2 µM fedratinib (FED-2), 3 µM fedratinib (FED-3), 5 µM fedratinib (FED-5), 5 nM VIC with 0.5 µM fedratinib (VIC-5 + FED-0.5), 5 nM VIC with 1 µM fedratinib (VIC-5 + FED-1), 5 nM VIC with 2 µM fedratinib (VIC-5 + FED-2), 5 nM VIC with 3 µM fedratinib (VIC-5 + FED-3), 5 nM VIC with 5 µM fedratinib (VIC-5+FED-5), or 0.1% DMSO (CON). Cell viability assay was performed as described in Section 4, Materials and Methods. The data are presented as the mean ±SD from two independent experiments repeated in triplicate. For co-treatments, significantly different at * *p* < 0.05 compared with the corresponding control. The “ns” is an abbreviation of “not significant”.

In order to measure P-gp protein levels, we performed Western blot analysis after 4 h or 24 h with 2 µM fedratinib treatment. There were no qualitative differences in P-gp expression levels between fedratinib treatment and DMSO-treatment control in both 4 h and 24 h (Figure 5A,B). It suggests that the mechanism of fedratinib results from the inhibition of P-gp activity, but not the repression of the transcription or translation of P-gp. Additionally, we have selected 1–2 µM fedratinib for use in the co-treatment experiments to determine the mechanisms of action (Figure 5C,D), since the concentrations worked best when combined with vincristine. This decision was also based on the observation that higher concentrations of fedratinib had cytotoxic effects on resistant KBV20C cells and resulted in only a small increase in the cytotoxic effect of VIC (Figure 5C,D).

## 3. Discussion

Drug repositioning or repurposing is the application of known drugs for new uses. It has been applied in the treatment of various diseases and has several advantages, including lower cost [10,12]. Given the lack of effective treatment strategies for MDR cancers, it is essential that we assess the suitability of repurposed drugs in this clinical field. We previously evaluated the cytotoxic effects of several repurposed drugs and suggested the application of anti-malarial, -retroviral, -histaminic, or -psychotic drugs and tyrosine kinase inhibitors as co-treatments of P-gp overexpressing drug-resistant cancer cells [32,39,40,41].

Various JAK2 inhibitors have been developed as treatments for leukemia, myelofibrosis, and myeloproliferative neoplasms [17,18]. Among the commercially available JAK2 inhibitors, three demonstrated cytotoxic effects in P-gp-overexpressing resistant cancer as part of combination therapies, including CEP-33779, NVP-BSK805, and XL019. These JAK2 inhibitors showed P-gp inhibitory activity and prevented P-gp-mediated efflux of anticancer drugs [19,20,21]. In this study, we tested an FDA-approved JAK2 inhibitor, fedratinib [22,23]. We found that VIC–fedratinib co-treatment increased cytotoxicity in P-gp-overexpressing drug-resistant cancer cells, which, barring further clinical trials, supports the application of fedratinib in patients resistant to antimitotic drugs. Our results were not limited to VIC co-treatment: we confirmed that fedratinib exhibited similar sensitization effects to eribulin, vinorelbine, and vinblastine co-treated resistant KBV20C cells. We hypothesize that fedratinib may be used in combination with various antimitotic drugs to sensitize resistant cancer cells.

VIC–fedratinib co-treatment reduced cellular proliferation and increased G2 arrest in resistant KBV20C cells. Through a quantitative annexin V analysis, we also demonstrated that VIC–fedratinib co-treatment increased early apoptosis in resistant KBV20C cells. Based on microscopic, FACS, and annexin V analyses, we concluded that early apoptosis was induced by fedratinib through increased G2 arrest and reduced proliferation in P-gp overexpressing resistant KBV20C cells.

By analyzing the expression of proteins involved in cellular signaling pathways, we found that DNA damaging-related marker pH2AX increased considerably in VIC–fedratinib co-treatment. According to Western blot analysis, p21 levels also increased in VIC–fedratinib co-treated cells, indicating that cell cycle arrest is driven by its upregulation. This is indicative of a mechanism involving G2 phase arrest via increased levels of DNA damage and p21 along with an increased number of cells undergoing early apoptotic death due to G2 arrest. Further in vivo studies using animal models or tumor spheroid assays should facilitate the application of fedratinib as a co-treatment with antimitotic drugs in patients who are resistant to combination therapies.

As the efflux of antimitotic drugs by P-gp is the main mechanism of resistance in KBV20C cells, we tested whether sensitization of VIC–fedratinib co-treatment resulted from the inhibitory activity of fedratinib. Fedratinib at a lower dose demonstrated similar P-gp-inhibitory activity to the well-studied P-gp inhibitor verapamil, suggesting that VIC–fedratinib sensitization results from the inhibitory activity of fedratinib preventing the efflux of VIC. Based on these results, we assume that fedratinib binds directly to P-gp in drug-resistant KBV20C cancer cells to prevent drug efflux. In addition, comparatively low doses of fedratinib showed similar cytotoxic effects to the clinically established P-gp inhibitor verapamil [21,30,33], implying that fedratinib has a higher binding affinity. In future studies, in vitro assays should be used to determine whether fedratinib binds directly to and inhibits the activity of P-gp.

Various selective JAK2 inhibitors have been developed (Table 1). The mechanism of action underlying the P-gp inhibitory function of CEP-33779, NVP-BSK805, and XL019 has recently been described in an in vitro model of P-gp-overexpressing drug-resistant cancer cells [19,20,21]. Molecular docking models showed that both CEP-33779 and NVP-BSK805 exhibited a higher predicted affinity for P-gp via direct binding [19,21]. Therefore, we compared the cytotoxic effects of fedratinib with CEP-33779 and NVP-BSK805. Fedratinib, CEP-33779, and NVP-BSK805 showed similar cytotoxic effects at the same dose in VIC-treated KBV20C cells, suggesting that each can function as a P-gp inhibitor. We also found similar degrees of early apoptosis between KBV20C cells treated with VIC–fedratinib, VIC–CEP-33779, and VIC–NVP-BSK805, indicating that co-treatment with JAK2 inhibitors can increase cytotoxicity in P-gp overexpressing resistant cancer cells by inducing early apoptosis. Notably, VIC–fedratinib, VIC–CEP-33779, and VIC–NVP-BSK805 exhibited similar sensitization mechanisms in P-gp-overexpressing resistant cancers. The different structures of JAK2 inhibitors can allow for their use in clinics as alternative backup inhibitors for resistant cancer cells. Our findings on JAK2 inhibitors could be useful in the development of effective regimens for cancer patients with drug-resistance. Considering that fedratinib monotherapy induces cytotoxicity in resistant KBV20C and sensitive parent KB cells to a similar degree, we propose that single treatments of fedratinib at low doses could be effective in drug-resistant cancers, though further research is required.

In conclusion, our study indicates that co-treatment of drug-resistant P-gp-overexpressing KBV20C cells with antimitotic drugs and the JAK2 inhibitor fedratinib can effectively induce cytotoxicity. Given its approval by the FDA and the consistent findings of our study, fedratinib holds considerable potential for application in the pharmacological treatment of antimitotic drug-resistant cancers.

## 4. Materials and Methods

### 4.1. Reagents and Cell Culture

VIC, vinorelbine, and vinblastine were purchased from Enzo Life Sciences (Farmingdale, NY, USA). Verapamil and Rhodamine123 (Rhodamine) were purchased from Sigma-Aldrich (St. Louis, MO, USA). Fedratinib, CEP-33779, NVP-BSK805, aripiprazole, and reserpine were purchased from Selleckchem (Houston, TX, USA). Eribulin (Eisai Korea, Seoul, South Korea) were obtained from the National Cancer Center in South Korea.

Antibodies against C-PARP and P-gp (antibody 1; #13342) were obtained from Cell Signaling Technology (Danvers, MA, USA). Antibodies against Cyclin D1, CDK2, P-gp (antibody 2; SC-55510), and GAPDH were obtained from Santa Cruz Biotechnology (Santa Cruz, CA, USA). Antibody against pH2AX was obtained from Sigma-Aldrich (St. Louis, MO, USA). Antibodies against α-LC3B, CDK1, and p21 were obtained from Abcam (Cambridge, U.K.).

Human oral squamous carcinoma multidrug-resistant KBV20C cell line, and its sensitive parent KB subline were obtained from Dr. Yong Kee Kim (College of Pharmacy, Sookmyung Women’s University, Seoul, South Korea) and have been previously described [32,42,43]. They were cultured in RPMI 1640 containing 10% fetal bovine serum, 100 U/mL penicillin, and 100 µg/mL streptomycin (WelGENE, Daegu, South Korea).

### 4.2. Microscopic Observation

Cellular proliferation and growth by microscope were observed as previously described [25,26,30]. Cells were grown in 60 mm diameter dishes and treated with fedratinib, verapamil, CEP-33779, NVP-BSK805, or 0.1% DMSO (control), alone and in combination with antimitotic drugs for 24 h or 48 h. The cells were then examined immediately in two independent experiments using an ECLIPSETs2 inverted routine microscope (Nikon, Tokyo, Japan) with a ×40 or a ×100 objective lens. They were qualitatively observed and confirmed in at least two independent experiments.

### 4.3. Cell Viability Assay

Cell viability tests were measured by a colorimetric assay using the EZ-CyTox cell viability assay kit (Daeillab, South Korea) according to the manufacturer’s instructions and as previously described [39,40,41]. Briefly, KB and KBV20C cells were grown in wells of 96-well plates and treated with 2 µM fedratinib, 5 µM fedratinib, 10 µM fedratinib, or 0.1% DMSO (control), alone and in combination with vincristine for 48 h. The cells were then incubated with EZ-CyTox solution for 1 h at 37 °C. Absorbance at 450 nm was measured using the VERSA MAX Microplate Reader (Molecular Devices Corp., Sunnyvale, CA, USA). All experiments were performed at least in triplicate and repeated twice.

### 4.4. Fluorescence-Activated Cell Sorting (FACS) Analysis

FACS analysis was performed as previously described [25,26,30]. Cells were grown in 60 mm diameter dishes and with 1 µM fedratinib, 2 µM fedratinib, or 0.1% DMSO (control), alone and in combination with antimitotic drugs for 24 h. The cells were then dislodged and pelleted by centrifugation. The pelleted cells were washed with PBS and suspended in 75% ethanol for at least 24 h at −20 °C. They were then washed with PBS again, and re-suspended in a cold propidium iodide (PI) staining solution (100 µg/mL RNase A and 50 µg/mL PI in PBS) for 30 min at 37 °C. The stained cells were analyzed in two independent experiments for relative DNA content using a Guava EasyCyte Plus Flow Cytometer (Merck Millipore, Burlington, MA, USA). They were qualitatively observed and confirmed in at least two independent experiments.

### 4.5. Annexin V Analysis

Annexin V analysis was performed by using the annexin V-fluorescein isothiocyanate (FITC) staining kit (BD Bioscience, Franklin, NJ, USA) as previously described [25,26,30]. Cells were grown in 60 mm diameter dishes and treated with fedratinib, CEP-33779, NVP-BSK805, or 0.1% DMSO (control), alone and in combination with vincristine for 24 h. The cells were then dislodged and pelleted by centrifugation. The pelleted cells were washed thoroughly with PBS. Cells in 100 µL of binding buffer received 5 µL of Annexin V-FITC and 5 µL of PI and were then incubated for 30 min at 20 °C. The stained cells were analyzed in two independent experiments using a Guava EasyCyte Plus Flow Cytometer (Merck Millipore, Burlington, MA, USA). They were qualitatively observed and confirmed in at least two independent experiments.

### 4.6. Rhodamine Uptake Tests

The tests used to assess the ability of a drug to inhibit P-gp were based on a previously described method [30,32,39]. Briefly, cells grown in 60 mm diameter dishes were treated with 10 µM verapamil, 2.5 µM aripiprazole, 5 µM reserpine, 2 µM fedratinib, or 0.1% DMSO (control) and then incubated for 1 h at 37 °C. The medium was removed, and the cells were washed thoroughly with PBS. The stained cells for 3 h were analyzed in two independent experiments using a Guava EasyCyte Plus Flow Cytometer (Merck Millipore, Burlington, MA, USA). They were qualitatively observed and confirmed in at least two independent experiments.

### 4.7. Western Blot Analysis

Western blotting analysis was performed as previously described [30,32,44]. Briefly, cells grown in 60 mm dishes were treated with fedratinib alone or in combination with vincristine for 24 h. The cells were then washed with cold PBS and detached by trypsin. For total protein extraction, cells were suspended in PRO-PREP™ protein extract solution (iNtRON, Seongnam, Korea) and placed on ice for 30 min. The suspension was collected after centrifugation at 10,000× *g* for 10 min at 4 °C. Protein concentrations were measured by using a protein assay kit (Bio-Rad, Hercules, CA, USA) according to the manufacturer’s instructions. The proteins were resolved by sodium dodecyl sulfate-polyacrylamide gel electrophoresis (SDS-PAGE) and subjected to Western blot analysis as previously described [30,32,44]. They were qualitatively observed and confirmed in at least two independent experiments.

### 4.8. Statistical Analysis

All data are presented as the mean ±S.D. from two independent experiments repeated in triplicate. Statistical analysis was performed using one-way analysis of variance and analysis of variance followed by Bonferroni’s test. Analysis was performed using GraphPad Prism software (version 5.0; GraphPad Software, San Diego, CA, USA). Statistical significance was set at *p* < 0.05.

## Figures and Tables

**Table 1:** JAK2 inhibitors and their P-gp inhibitory activity.

JAK2 Inhibitors	Clinical Phase (Year)	P-gp-Inhibitory Activity (Year)
CEP-33779	Mouse model (2011)	Yes (2014)
NVP-BSK805	Mouse model (2020)	Yes (2017)
XL019	Phase I (2014)	Yes (2017)
Pacritinib	Closer to FDA-approved (2021)	Yes (In press)
Fedratinib	FDA-approved (2019)	Yes (In this study)
Ruxolitinib	FDA-approved (2011)	Not determined
Baricitinib	FDA-approved (2018)	Not determined
Momelotinib	Fast track designation by FDA (2019)	No (In press)
Gandotinib	Phase II (2021)	Not determined
AZD1480	Phase I (2015)	Not determined
NS-018	Phase I/2 (2017)	Not determined
TG101209	Mouse model (2013)	Not determined
FLLL32	Mouse model (2010)	Not determined
AT9283	Mouse model (2009)	Not determined
WP1066	Mouse model (2010)	No (In press)
AG-490	Mouse model (2002)	Not determined
AZ960	Cell lines (2009)	Not determined
TG89	Cell lines (2019)	Not determined
NSC 42834 (Z3)	Cell lines (2008)	Not determined

## Abbreviations

MDR	Multidrug resistance
P-gp	P-glycoprotein
JAK2	Janus kinase 2
FED	Fedratinib
CEP	CEP-33779
NVP	NVP-BSK805
VIC	Vincristine
ERI	Eribulin
VIO	Vinorelbine
VIB	Vinblastine
VER	Verapamil
DMSO	Dimethylsulfoxide
C-PARP	Cleaved ploy ADP ribose polymerase
FACS	Fluorescence-activated cell sorting
FDA	The United States Food and Drug Administration
